# Human Genetic Susceptibility to Native Valve *Staphylococcus aureus* Endocarditis in Patients With *S. aureus* Bacteremia: Genome-Wide Association Study

**DOI:** 10.3389/fmicb.2018.00640

**Published:** 2018-04-04

**Authors:** Karen Moreau, Alisson Clemenceau, Vincent Le Moing, David Messika-Zeitoun, Paal S. Andersen, Niels E. Bruun, Robert L. Skov, Florence Couzon, Coralie Bouchiat, Marie L. Erpelding, Alex van Belkum, Yohan Bossé, Xavier Duval, Francois Vandenesch, The French VIRSTA-AEPEI, COFRASA Study Groups, The Danish DANSAB Study Group

**Affiliations:** ^1^International Center for Infectiology Research, CNRS UMR5308, INSERM U1111, Ecole Normale Supérieure de Lyon, Université Lyon 1, Lyon, France; ^2^Institut Universitaire de Cardiologie et de Pneumologie de Québec – Université Laval, Quebec City, QC, Canada; ^3^Department for Infectious Diseases and Tropical Medicine, Centre Hospitalier, Universitaire de Montpellier, Montpellier, France; ^4^Department of Cardiology, AP-HP, Bichat Hospital, Paris, France; ^5^INSERM U698 and University Paris 7, Paris, France; ^6^Statens Serum Institut, Copenhagen, Denmark; ^7^Clinical Institute, Aalborg University, Aalborg, Denmark; ^8^Centre National de Référence des Staphylocoques, Centre de Biologie Est, Hospices Civils de Lyon, Lyon, France; ^9^INSERM, CIC-1433 Clinical Epidemiology, CHRU Nancy, University of Lorraine, Nancy, France; ^10^Data Analytics Unit, bioMerieux, La Balme-les-Grottes, France; ^11^Département de Médecine Moléculaire, Université Laval, Quebec City, QC, Canada; ^12^INSERM, UMR1138 IAME, CIC 1425, Université Paris Diderot, Paris, France; ^13^AP-HP, Hôpital Bichat-Claude Bernard, Paris, France

**Keywords:** infectious endocarditis, *Staphylococcus aureus*, GWAS, bacteremia, SLC7A14

## Abstract

*Staphylococcus aureus* infective endocarditis (SaIE) is a severe complication of *S. aureus* bacteremia (SAB) occurring in up to 22% of patients. Bacterial genetic factors and host conditions for SaIE have been intensely studied before; however, to date no study has focused on predisposing host genetic factors to SaIE. The present study aimed to identify genetic polymorphisms associated with SaIE by a Genome-Wide Association Study (GWAS) of 67 patients with definite native valve SaIE (cases) and 72 matched native valve patients with SAB but without IE (controls). All patients were enrolled in the VIRSTA cohort (Le Moing et al., 2015) study. Four single nucleotide polymorphisms (SNPs) located on chromosome 3 were associated with SaIE (*P* < 1 × 10^-5^) without reaching conventional genome-wide significance. For all, the frequency of the minor allele was lower in cases than in controls, suggesting a protective effect of the minor allele against SaIE. The same association was observed using an independent Danish verification cohort of SAB with (*n* = 57) and without (*n* = 123) IE. *Ex vivo* analysis of aortic valve tissues revealed that SaIE associated SNPs mentioned above were associated with significantly higher mRNA expression levels of SLC7A14, a predicted cationic amino acid transporter protein. Taken together, our results suggest an IE-protective effect of SNPs on chromosome 3 during the course of SAB. The effects of protective minor alleles may be mediated by increasing expression levels of SLC7A14 in valve tissues. We conclude that occurrence of SaIE may be the combination of a well-adapted bacterial genotype to a susceptible host.

## Introduction

*Staphylococcus aureus* is the second most frequent pathogen causing bloodstream infection with an average rate of 25 per 100,000 individuals per year in North America and western European countries (Laupland, 2013). Infective endocarditis (IE) is one of the most severe complications of *S. aureus* bloodstream infection (SAB) and occurs in 5–22% of SAB cases (del Rio et al., 2009; Rasmussen et al., 2011). Hence, *S. aureus* is nowadays the most prevalent bacterial species responsible for IE in the majority of developed countries (Murdoch et al., 2009; Duval et al., 2012; Selton-Suty et al., 2012). Fortunately, IE is still a rare but severe disease, with a mortality of approximately 20% [10–30%] during the initial hospitalization phase (Moreillon and Que, 2004; Murdoch et al., 2009; Hoen and Duval, 2012), and up to 40% at a 5 year endpoint (Bannay et al., 2011). Therefore, detecting and identifying the pathogen and potential host determinants of *S. aureus* IE (SaIE) in the course of SAB is of major importance for management and prevention of infectious endocarditis.

Several IE risk factors related to the patients’ characteristics have been described including male sex (Moreillon and Que, 2004; Erichsen et al., 2016) with no particular explanation, history of IE, congenital heart disease (especially cyanogen corrected) (Takeda et al., 2005), or presence of intra-cardiac devices, mainly prosthetic valves. Regarding host-related factors in the context of SAB, underlying diseases such as diabetes, HIV infection, hemodialysis and intravenous drug use have been shown to be primary SaIE risk factors (Li and Somerville, 1998; Cabell et al., 2002; Chang et al., 2003; Miro et al., 2005; Gebo et al., 2006; Habib et al., 2009; Hoen and Duval, 2012). Our group recently developed and validated a simple score-based prediction rule to quantify the risk of SaIE within 48 h after SAB diagnosis in patients with community-acquired (CA) or healthcare-associated (HA) SAB, using the largest prospective cohort of SAB patients reported to date (the VIRSTA cohort) (Le Moing et al., 2015; Tubiana et al., 2016). Note that 30–50% of SaIE occurs without any obvious involvement of the classical host risk factors, suggesting that additional host genetic factors may be involved (Hoen and Duval, 2012; Le Moing et al., 2015).

A variety of research findings suggest that there is a genetic basis for human susceptibility to *S. aureus* colonization and/or infection. Evidences include: (i) genetic polymorphism in various loci associated with nasal carriage (Emonts et al., 2008; Ruimy et al., 2010), (ii) higher rates of *S. aureus* infections in distinct ethnic populations (Maori and Pacific island people versus European; Aboriginal Australian versus Australian, American black versus white American) (Embil et al., 1994; Maguire et al., 1998; Hill et al., 2001; Klevens et al., 2007; Kallen et al., 2010), (iii) familial clusters of *S. aureus* infection (Oestergaard et al., 2016); (iv) rare genetic conditions associated with susceptibility to *S. aureus* (Medvedev and Vogel, 2003; Picard et al., 2003). More recently, genome-wide association study (GWAS) has been successful to identify genetic variants robustly associated with human diseases (Hindorff et al., 2009). Three GWAS have investigated potential associations between common genetic variants and human susceptibility to *S. aureus* infection. Nelson et al. (2014) compared 361 cases of *S. aureus* bacteremia to 699 controls (Nelson et al., 2014). Ye et al. (2014) compared 309 infected people to 2952 controls (Ye et al., 2014). In both cases, no genome-wide significant common variant was found to be associated with risk of acquiring *S. aureus* infection. Most recently, DeLorenze et al. (2016) presented the first GWAS evidence of human genetic susceptibility to *S. aureus* infection (DeLorenze et al., 2016). The investigators genotyped a Caucasian population of 4,701 cases of *S. aureus* infection and 45,344 matched controls. Two imputed SNPs (rs115231074: *p* = 1.3 × 10^-10^ and rs35079132: *p* = 3.8 × 10^-8^) achieved genome-wide significance, and one adjacent SNP was nearly significant genomewide (rs4321864: *p* = 8.8 × 10^-8^). These polymorphisms were located near HLA-DRA and HLA-DRB1 genes on chromosome 6 in the HLA class II region. These results were reinforced in an admixture mapping study identifying the HLA class II region on chromosome 6 associated with SAB susceptibility (Cyr et al., 2017). However, to date no GWAS has been conducted for SA-IE in the course of bacteremia.

In the present study, we conducted a GWAS to identify genetic polymorphisms associated with SaIE in 67 patients presenting definite native valve IE (cases) and 72 matched patients with SAB (controls) enrolled in the VIRSTA cohort study. A replication study was then performed using an independent Danish cohort of patients with SAB, with and without IE.

## Materials and Methods

### Study Participants of the French VIRSTA Cohort

Patients were enrolled as part of the French national prospective multicenter cohort VIRSTA (Le Moing et al., 2015). Briefly, 2,091 patients aged over 18 years and presenting at least one peripheral blood culture positive for *S. aureus* were included from 2009 to 2011. Patients were recruited in eight teaching hospitals. Among these patients, 221 (11%) had definite IE according to the modified Duke criteria (Li et al., 2000). To study differences between IE and bacteremia isolates, this nested case-control study retained 211 patients with definite native valve IE and the presence of echocardiographic vegetation as defined cases. Patients with native cardiac valve meeting the “possible” or “excluded IE” definition, without any argument for IE using Transesophageal Echocardiography (TEE) during a 3-month follow-up period were defined as controls. Cases and controls were matched according to age, sex and the SAB setting of acquisition [healthcare-related (HA) either nosocomial or non-nosocomial vs. community-acquired (CA)]. All patients provided written informed consent.

Comparison of the characteristics of the patients enrolled in the present case control genetic study versus the entire cohort of the VIRSTA study was realized using Chi-square or Fisher’s exact test for qualitative variables and Student’s *t*-test for quantitative variables with SAS9.3.

The French national ethics committee “Comité de Protection des Personnes SudMéditerrannée IV” approved the study. The VIRSTA study is registered in the European Clinical Trials Database (EUDRACT) (number: 2008-A00680-55).

### DNA Extraction and Genotyping

DNA was extracted from blood samples using AGOWA DNA isolation kits (LGC) according to the manufacturer’s protocols and genotyped on the Illumina HumanOmni Exp-12v1 BeadChip array at the Human Genomics Facility (HuGeF^1^) at Erasmus University Medical Center, Rotterdam, The Netherlands (Prof. Dr. André Uitterlinden), according to the manufacturer’s protocols.

A genotyping report was produced in GenomeStudio V2011.1. Low quality SNPs were removed based on the Illumina GenCall (<0.1), call rate lower than 97%, Hardy–Weinberg equilibrium deviations with *p* < 7 × 10^-8^, minor allele frequencies (MAF) smaller than 0.01 or being monomorphic loci. Low quality samples were removed based on a SNP call rate <95%, sex discrepancies and genetic background outliers using Eigenstrat with HapMap subjects as internal controls (Price et al., 2006). Quality controls were performed in R 3.4.2 statistical software and PLINK, an open-source whole genome association analysis toolset (Purcell et al., 2007). Association between genotypes and IE was evaluated using chi-square tests implemented in PLINK. The genome-wide significant *p*-value cut-off was set at an established standard (*p*-value < 5 × 10^-8^) (Pe’er et al., 2008). We set the level of “suggestive” significance at a *p*-value lower or equal to 1 × 10^-5^. The linkage disequilibrium (LD) plot for SNPs on chromosome 3 was produced with Haploview 4.2 (Barrett et al., 2005).

### Minor Allele Frequencies of rs2287489 and rs4955730 in Control and Reference Populations

To assess the frequency of alleles of interest in the general population which were of similar geographic origin as the VIRSTA cohort, we used a cohort of subjects not infected with *S. aureus*: the COFRASA cohort consisting of 486 patients with degenerative aortic valve stenosis (Guauque-Olarte et al., 2015; Nguyen et al., 2015). Inclusion criteria were, at least, mild aortic stenosis (mean pressure gradient ≥ 10 mm Hg) and aortic valve structural changes (thickening/calcification). Exclusion criteria were aortic valve stenosis due to rheumatic disease or radiotherapy, ongoing or previous aortic endocarditis, coexisting aortic regurgitation, other valvular diseases and severe respiratory or renal insufficiency (creatinine clearance ≤ 30 ml/min). DNA was extracted from frozen buffy coat using the QIAamp^®^ DNA Blood Maxi kit (QIAGEN). Genotyping was performed at the McGill University and Genome Quebec Innovation Center using the Illumina HumanOmniExpress-12v1.0 BeadChip. Quality controls for SNPs and samples were performed as described above. Analyses were conducted to determine the minor allele frequencies of rs2287489 and rs4955730. The allele frequencies of these two SNPs were also compared to publically available databases including the Exome Aggregation Consortium (ExAC) and the Genome Aggregation Database (gnomAD) (Lek et al., 2016), the 1000 Genomes Project (1000 Genomes Project Consortium et al., 2015) and the Human Longevity Inc (HLI) 10,000 genomes (Telenti et al., 2016).

COFRASA participants were enrolled under procedures approved by the “Comité de Protection de Personnes”, Hotel Dieu, Paris, France. The procedure is registered in clinicalTrial.gov number NCT 00338676.

### Replication Set: The DANSAB Danish Cohort

To validate the results obtained with the VIRSTA cohort, analysis of the top SNPs was carried out using the DANSAB Danish cohort. The DANSAB cohort is a subset of individuals partaking in a large continuous study, running since 2009, involving *S. aureus* bacteremia patients from six large clinical microbiology departments (DANSAB study). The patients selected in this study were defined as patients with *S. aureus* bacteremia. They were divided into two groups: (1) a control group (*N* = 144) with bacteremia with no secondary infections up to a year after initial diagnosis of *S. aureus* bacteremia and (2) a group with definite IE according to Duke’s criteria (*N* = 57). All patients were > 18 years of age and suffered from either CA or HA related infections. Of these, we were able to obtain genotyping data on 123 controls and 57 IE cases.

Samples were obtained through either collection of blood cultures with DNA purified from between 5 and 10 mL blood culture using a Chemagic Star DNA Blood10K kit (Perkin Elmer) or from the Copenhagen Biobank with DNA purified using the QiaAMP Blood Midikit (Qiagen, Germany).

The regions encompassing the candidate SNPs, namely rs2287489 and rs4955730 were amplified by PCR using Gotaq kit (Promega) and the forward (5′-CAGAGCGGGGGTCTGCTTAC-3′) and reverse (5′-TTTCGTGGCCAGGCCACCAC-3′) primers for rs4955730 or forward (5′-CTGGGCTGAGAGAGGGGGC-3′) and reverse (5′-TTGTACAGCACACCAATGGGA-3′) primers for rs2287489. Primers were designed on SLC7A14 gene sequence (accession number NM_020949). The DNA sequences were obtained by Sanger sequencing and genotypes at both SNPs were read from the chromatograms.

DANSAB participants were enrolled under procedures approved by the Danish Regional Ethics Committee (journal no. H-4-2014-132) and the Danish Data Protection Agency (GEH2014-053 // I-suite no 03372 and journal no. 2007-58-0015).

### Valve Expression Quantitative Trait Loci (eQTL)

The functional characterization of SaIE-associated SNPs was extended to genotype-specific gene expression analysis in human aortic valves. Tricuspid aortic valves were explanted from 24 white male patients who underwent aortic valve replacement for severe calcific aortic valve stenosis at the Institut Universitaire de Cardiologie et de Pneumologie de Québec (IUCPQ). Only non-rheumatic aortic valves with the same fibro-calcific remodeling score were selected (Warren and Yong, 1997). Patients with other valve diseases, previous cardiac surgery, and moderate or severe aortic regurgitation were excluded. DNA was extracted from 100 mg of frozen aortic valve tissue digested with Qiagen’s proteinase K at 56°C for 16 h, followed by column purification using QIAamp^®^ DNA Mini kit (Qiagen). DNA quality was assessed by UV_260/280__nm_ ratio. DNA concentration was determined by QuantiT^TM^ PicoGreen^TM^ dsDNA Assay Kit. Genotyping was performed using the Illumina Infinium HumanOmni2.5–8 BeadChip. SNPs were excluded based on Hardy–Weinberg (*P* < 0.01), minor allele frequency smaller than 0.01 or call rates lower than 0.97. DNA samples were removed based on completion rate lower than 0.95, sex discrepancies, duplicates and genetic background. Data from 24 patients and 1,474,616 SNPs passed all these quality controls and were available for analysis. Total RNA was extracted from 100 mg of tissue using RNeasy^®^ Plus Universal Mini Kit (Qiagen). RNA quality was assessed by the Agilent 2100 Bioanalyzer system. RNA concentration was determined by UV_260__nm_. Gene expression was measured on the Illumina HumanHT-12v4 Expression BeadChip. Samples were removed based on outliers in pairwise correlation, principal components analysis (PCA), and hierarchical clustering. After these quality controls, 22 samples were available for analysis. mRNA expression data were log2 transformed and quantile normalized using the lumi package in R (Du et al., 2008). Both whole-genome genotyping and gene expression were performed in a single batch at the McGill University and Genome Quebec Innovation Center. mRNA transcripts were selected for valve eQTL analysis if they were located near the top GWAS SNPs. Association testing between genotypes and mRNA expression levels was performed in PLINK using the –assoc command (Wald test). Written informed consent was obtained from all study participants and the study was approved by the ethics committee of the IUCPQ.

## Results

### SNPs Potentially Associated With SaIE

**FIGURE 1:**
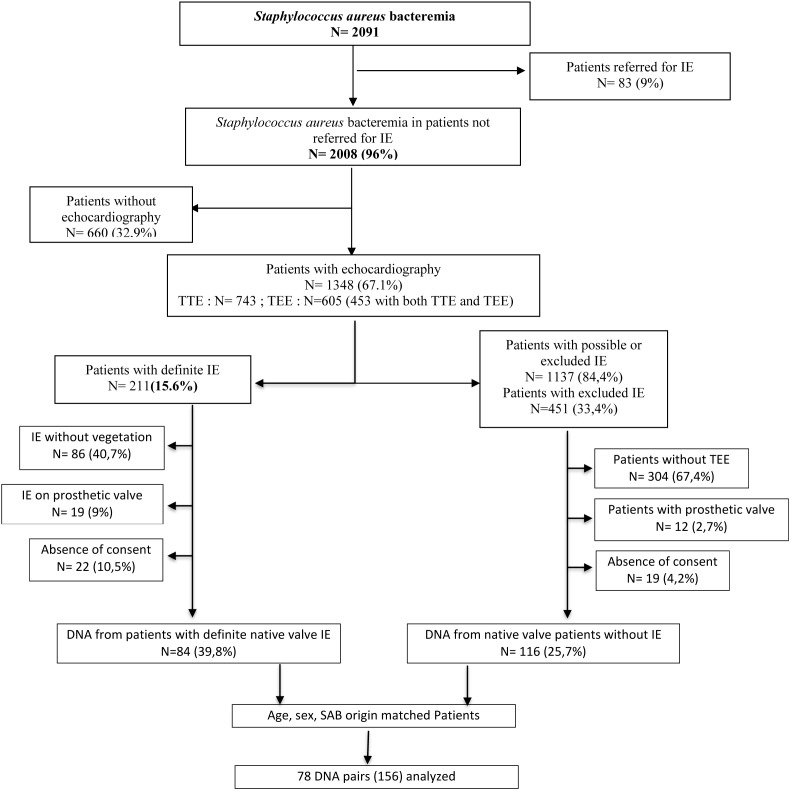
Flow chart of patient recruitment in the VIRSTA case control study. IE, Infective endocarditis. TEE, Transesophageal echocardiography. TTE, Transthoracic echocardiography.

Among the 2,091 patients enrolled in the VIRSTA cohort study (Le Moing et al., 2015), 156 patients fulfilled the inclusion criteria for the case control study, gave consent and were genotyped by SNP microarray (**Figure 1**). Only patients with native valves were retained considering that the presence of a prosthetic valve is a major risk factor as such and that may mask the host genetic risk factors. Eventually, 78 patients presenting definite IE were compared to 78 SAB –age, -sex and -origin (healthcare associated vs. community acquired) matched control patients presenting a SAB (**Figure 1**).

**Table 1** shows the characteristics of the 78 case-patients enrolled in the case control study as compared to the 106 patients with definite native valve SaIE from the whole cohort VIRSTA study 2009–2011. The only difference between the two groups was the mortality, which was higher in patients who were not enrolled in the case-control study probably because the severity of their disease precluded the gathering of the consent for DNA collection.

**Table 1:** Comparison of the characteristics of the patients enrolled in the case control genetic study and in the whole cohort.

	Definite *Staphylococcus aureus* native valve endocarditis			SAB patients without IE		
Characteristics	Cases	Whole VIRSTA cohort	*p*	Controls	Whole VIRSTA cohort	*p*
*N*	78	106		78	1718	
Male *n* (%)	52 (66)	66 (64)	0.74	52 (66)	1106 (64)	0.80
Mean age (years)	58	61	0.26	58	66	0.0003
Injecting drug use *n* (%)	12 (15)	18 (17)	0.98	6 (8)	33 (2)	0.006
Immuno-suppression^∗^	25 (32)	28 (27)	0.49	19 (24)	652 (38)	0.01
Methicillin resistance	12 (15)	9 (9)	0.17	6 (8)	347 (20)	0.006
Setting of acquisition *n* (%)			0.67			<0.0001
Community	47 (60)	63 (60)		52 (67)	367 (21)	
Healthcare-related	12 (15)	19 (18)		8 (10)	306 (18)	
Nosocomial	19 (24)	21 (20)		18 (23)	989 (58)	
Severe sepsis	36 (46)	49 (47)	0.93	13 (17)	390 (23)	0.19
Death at week 12	19 (24)	47 (45)	0.003	4 (5)	563 (33)	<0.0001

*VIRSTA study 2009-2011. ^∗^Patient was classified as suffering immunodepression if presenting a primary immune deficiency, or a solid cancer, leukemia or lymphoma, or HIV infection with CD4 cell count < 200/ml, or was treated with corticosteroids or other immunosuppressive therapy. The numbers in brackets represent the percentage of patients.*

**Table 1** also shows the characteristics of the 78 control patients enrolled in the case-control genetic study as compared to the 1,718 patients without SaIE from the whole cohort VIRSTA study 2009–2011. Differences between controls and other patients with bacteremia without endocarditis were more pronounced and probably mostly due to the matching of controls with cases which were mostly community-acquired while patients enrolled in the VIRSTA cohort comprised a large proportion of healthcare-related bacteremia. In addition, the presence of a prosthetic valve was excluded in the case control genetic study.

**FIGURE 2:**
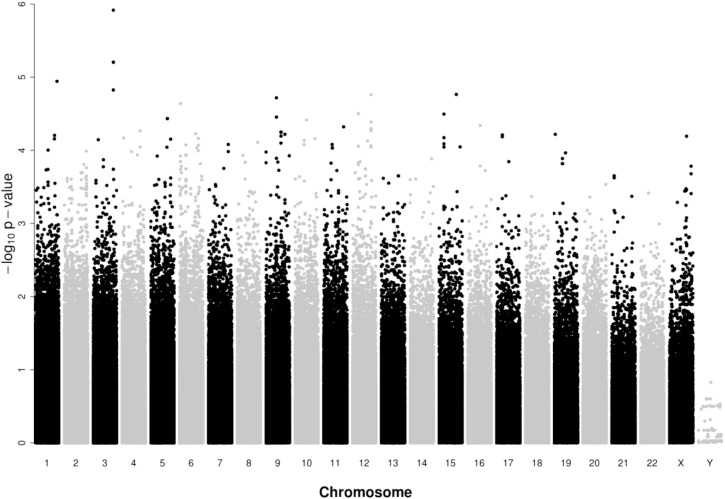
Manhattan plot of genome-wide association results for the discovery VIRSTA cohort. The *x*-axis represents the chromosome number and the *y*-axis shows the *P*-values in – log10 scale. Dot represent SNP. A total of 67 cases and 72 controls are compared. 631,710 SNPs that passed all quality control filters are presented. As indicated in **Table 2**, two SNPs on chromosome 3 have the same *p*-value; these two points overlap in present figure.

**FIGURE 3:**
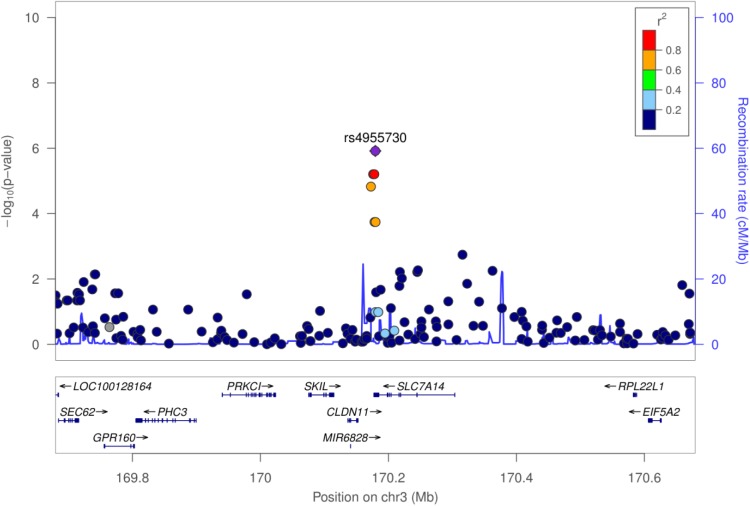
Regional association plot of the SaIE susceptibility locus on chromosome 3. The *y* axis shows the *P*-values in –log10 scale for SNPs up- and downstream of the sentinel SNP (purple dot). The extent of linkage disequilibrium (LD; *r*^2^ values) for all SNPs with the sentinel SNP is indicated by colors. The location of genes is shown at the bottom.

**FIGURE 4:**
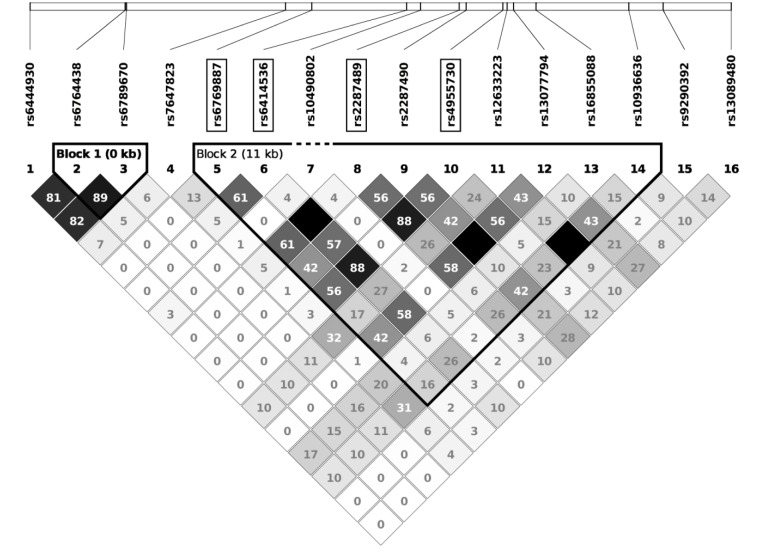
Linkage disequilibrium plot for SNPs located near (±10 kb) rs2287489 and rs6414536 in the VIRSTA cohort. The white horizontal bar (top) illustrates SNPs location on a physical scale. LD values (*r*^2^) are presented in boxes. Strong LD is defined as an *r*^2^ > 0.8 (darkest color). The top four associated SNPs are framed.

From the 716,503 SNPs and 156 samples available, a total of 84,793 SNPs and 17 samples failed quality controls, leaving 631,710 SNPs and 139 participants (67 cases and 72 controls) for analysis. A Manhattan plot of the GWAS results on SaIE is presented in **Figure 2**. The Q–Q plot is shown in Supplementary Figure 1. No SNPs were significantly associated with SaIE at the genome-wide level (*p*-value < 5 × 10^-8^). Four “suggestive” SNPs (*p*-value < 1 × 10^-5^) were located on one locus on chromosome 3, near the genes CLDN11 and SLC7A14 (**Figure 3**). **Table 2** shows the minor allele frequencies for cases and controls for these four three SNPs as well as odd ratios. For these four SNPs, the frequency of the minor allele was lower in cases than in controls, suggesting a protective effect of the minor allele against SaIE. The top three associated SNPs (rs6414536, rs2287489, and rs4955730) are in high LD (*r*^2^ > 0.88). The fourth SNP (rs6769887) located close to the top 3 associated SNPs and in moderate LD (*r*^2^ > 0.56) had a *p*-value of 1.5 × 10^-5^. A LD plot for all SNPs located near (±10 kb) rs2287489 and rs6414536 is presented in **Figure 4**.

**Table 2:** Genetic variants suggestively associated with *S. aureus* infective endocarditis in the GWAS of 67 cases and 72 controls of the discovery VIRSTA cohort.

SNP	Chromosome	Position on Hg19	A1/A2	A1 in cases	A1 in controls	OR	*P*-value	Position to SLC7A14 gene	Position to CLDN11 gene
rs6414536	3	170176173	A/G	0.127	0.361	0.257	6.248e-06	–	Intron
rs2287489	3	170178057	A/C	0.127	0.361	0.257	6.248e-06	3′ UTR	Intron
rs4955730	3	170179621	G/A	0.112	0.361	0.223	1.212e-06	3′ UTR	Intron
rs6769887	3	170172774	G/A	0.082	0.285	0.225	1.496e-05	–	Intron

*Positions of the 4 SNPs on chromosome 3 and relative to the SLC7A14 and CLDN11 genes are indicated. A1, minor allele; A2, major allele; OR, Odd Ratio. Hg19, human genome 19.*

### Comparison to Control and Reference Populations

The frequency of minor alleles in our cases and control populations was compared to the allele frequencies in a control population similar in time and space to the discovery VIRSTA cohort population. For this purpose, we used the COFRASA cohort involving 486 French patients with degenerative aortic valve stenosis (Guauque-Olarte et al., 2015; Nguyen et al., 2015). The frequencies of minor alleles for the top two SNPs (rs2287489 and rs4955730, rs6414536 was dropped as it is in perfect LD with rs2287489) were 0.24 in the COFRASA population, which is consistent with previously published studies of reference populations (1000 Genomes Project Consortium et al., 2015; Lek et al., 2016; Telenti et al., 2016). In our population of cases, the frequency of the minor alleles is much lower (0.13 and 0.11 for the two SNPs respectively) than the general population. Conversely, the frequency of the minor alleles is higher in our population of controls (0.36 for both SNPs) compared to COFRASA and reference populations (**Table 3**). These results are however consistent with the hypothesis of a protective effect of the minor allele against SaIE.

**Table 3:** Minor allele frequencies of rs2287489 and rs4955730 in cases and controls from VIRSTA compared to COFRASA and reference populations.

SNP	A1/A2	A1 in 67 VIRSTA cases	A1 in 72 VIRSTA controls	A1 in 426 COFRASA patients	A1 in European reference populations^∗^
rs2287489	A/C	0.127	0.361	0.245	0.24–0.26
rs4955730	G/A	0.112	0.361	0.242	0.24–0.25

*A1, minor allele; A2, major allele. ^∗^(1000 Genomes Project Consortium et al., 2015; Lek et al., 2016; Telenti et al., 2016).*

### Replication Using an Independent Cohort (DANSAB)

The two GWAS top hits on chromosome 3 were then analyzed in an independent cohort of Danish patients presenting either a non-complicated bacteremia (controls) or SaIE (cases). We genotyped the top two SaIE-associated SNPs (rs2287489 and rs4955730) in DANSAB. Although the results are not statistically significant, the minor alleles were also underrepresented in the cases compared to the controls, suggesting a protective effect as observed in the GWAS of the French VIRSTA cases and controls (**Table 4**). The effect sizes were smaller than those observed in the VIRSTA cohort.

**Table 4:** Association analysis of two top SNPs on chromosome 3 and infection-induced endocarditis in an independent case-control series of patients with *S. aureus* endocarditis: the Danish DANSAB cohort.

SNP	A1/A2	A1 in 57 SaEI Cases	A1 in 123 controls	OR	*P*-value
rs2287489	A/C	0.2946	0.3208	0.8843	0.6215
rs4955730	G/A	0.2797	0.3171	0.8362	0.468

*A1, minor allele; A2, major allele; OR, Odd Ratio.*

### Impact of SaIE-Associated SNPs on Gene Expression

**FIGURE 5:**
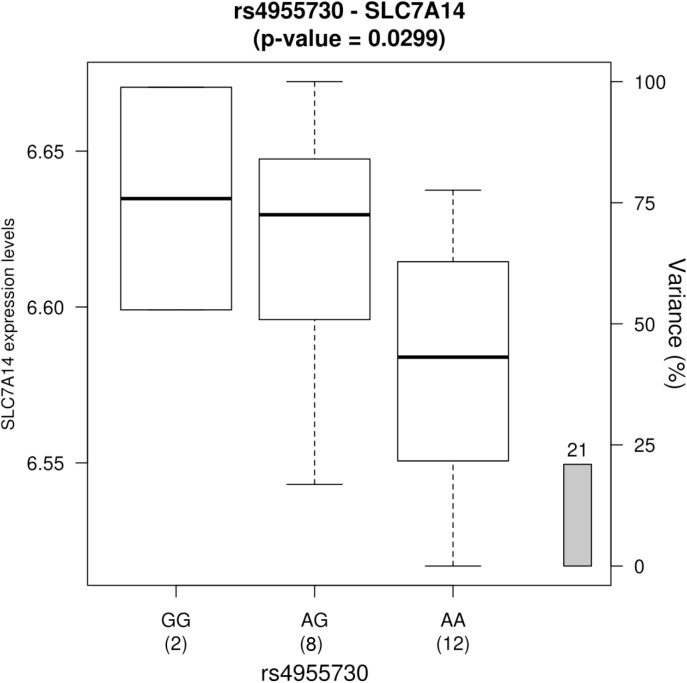
Gene expression levels of the SLC7A14 gene in human aortic valves according to 3 genotype groups for the SNP rs4955730 (*n* = 22, *p* = 0.0299). The left *y*-axis represents gene expression levels in the aortic valves. The *x*-axis represents the three-genotype groups for the SNP rs4955730 with the number of subjects in parenthesis. The right *y*-axis represents the percent variance (*r*^2^) in expression levels explained by the genotype for this SNP. The G allele, that seems to have a protective effect for SaIE, is associated with a higher level of gene expression.

Valve eQTL analyses were performed to evaluate whether the top GWAS SNPs were associated with mRNA expression levels of nearby genes in human aortic valves. No SNPs were associated with the expression of CLDN11 (data not shown). In contrast, significant associations were observed for SLC7A14. **Figure 5** shows result for the valve eQTL rs4955730-SLC7A14. The protective allele for SaIE (rs4955730-G) was associated with significantly increased mRNA levels of SLC7A14 (encoding a trans-membrane protein with putative amino acid transport function) in aortic valve tissues. These results suggest that the minor allele for rs4955730 decreases vulnerability to SaIE through up-regulation of SLC7A14 in valve tissue.

## Discussion

Although numerous risk factors related to the host have been identified, 30–50% of endocarditis occur in patients without any known risk factors (Hoen and Duval, 2012; Le Moing et al., 2015). Both host and bacterial genetic variation may explain the difference in IE susceptibility. Considering *S. aureus*, genetic variation of genes that encode for virulence, antibiotic resistance and host adaptation, may contribute to the occurrence of SaIE in the course of bacteremia. By analyzing the *S. aureus* isolates from the VIRSTA cohort, we recently demonstrated that the strains of *S. aureus* associated with endocarditis were genotypically distinct from those responsible for uncomplicated bacteremia (Bouchiat et al., 2015). We showed that the ability of *S. aureus* to cause endocarditis in the course of bacteremia was not linked to one or a few bacterial factors but more likely to a combination of more subtle genetic markers (Bouchiat et al., 2015).

To our knowledge, the present study is the first GWAS examining potential genetic predisposition for localization to the endocardium in case of *S. aureus* bacteremia and highlights four SNPs closely located on chromosome 3 suggestively associated with SaIE. The frequency of the minor allele was lowered in SaIE than in bacteremia controls, non-infected French patients of the COFRASA cohort, and published reference populations (1000 Genomes Project Consortium et al., 2015; Lek et al., 2016; Telenti et al., 2016). The protective effect of the two top SNPs toward the occurrence of SaIE was also observed in a second independent Danish cohort (DANSAB), however with noticeable differences in allele frequencies compared to the VIRSTA cohort. Both cases and controls from DANSAB displayed a higher frequency of the minor allele than cases and controls from VIRSTA respectively. This observation may result from sample bias or to differences at the level of the population structure among Danish people, which is markedly homogenous and genetically distinct from the French population structure (Athanasiadis et al., 2016). Indeed, the main limitation of the present study is the lack of statistical significance obtained in the GWAS, a method that is most successfully conducted using larger sample sizes. However, the fact that the protective effect of those three SNPs toward the occurrence of SaIE was observed in two independent cohorts suggests that the signal we observed is consistent. Larger cohorts will be needed to confirm our results but such studies will be very demanding.

The three SNPs are closely located on chromosome 3, in a region encompassing two genes encoding Claudin 11 and SLC7A14 proteins. Interestingly, in an *ex vivo* analysis of valve tissues, we showed that one of these SNPs was associated with significant up-regulation of SLC7A14, an orphan membrane-spanning protein (**Figure 5**). Taken together, our results suggest that the protective alleles mediate their effects by increasing the mRNA expression levels of SLC7A14 in valve tissues.

SLC7A14 (Solute Carrier Family 7, Member 14) is predicted to encode a glycosylated, cationic amino acid transporter protein with 14 trans-membrane domains. This gene is primarily expressed in skin fibroblasts, neural tissue, and primary endothelial cells, and its protein is predicted to mediate lysosomal uptake of cationic amino acids. Mutations in this gene are associated with autosomal recessive retinitis pigmentosa (Jin et al., 2014). As little is known about the function of SLC7A14 protein, it is difficult to speculate on the pathophysiological mechanism linking expression of this gene to the occurrence of SaIE. However, the valve eQTL analysis performed in this study provides the first biological clue about how genetic variants located on chromosome 3 could be associated with SaIE. Additional studies will be needed to confirm these observations and uncover the detailed mechanism of how these variants confer predisposition to endocarditis.

Previous studies attempted to identify genetic variants associated with infective endocarditis regardless of the etiologic agent. Analyzes were made on small cohorts of IE patients compared to healthy blood donors and targeted *a priori* candidates such as genes involved in inflammation. Polymorphisms in *TLR6, IL1B, IL12B, CRP* and *CALCR* genes but not *IL6* and *TNF* were associated with a decreased risk of IE (Golovkin et al., 2015; Ponasenko et al., 2017) while SNPs in *IL1B, IL6,TLR2 and TNF* genes were associated with IE (Bustamante et al., 2011; Giannitsioti et al., 2014; Weinstock et al., 2014) as compared to healthy donors. None of these SNPs were identified in the present study, perhaps because our study focused on *S. aureus* only and our comparator group was *S. aureus* bacteremia patients.

## Conclusion

The transition from uncomplicated *S. aureus* bacteremia to infective endocarditis –which remains the most pressing medical issue- likely results from a complex interplay between environmental factors, the host and the pathogen. The occurrence of SaIE may be the combination of a well-adapted bacterial genotype to a susceptible host, susceptibility which may be genetically-determined in some cases of native valve endocarditis.

## Data Availability

The raw data supporting the conclusions of this manuscript will be made available by the authors, without undue reservation, to any qualified researcher.

## Membership of the Virsta, Cofrasa and Dansab Study Groups

### The VIRSTA Study Group

Clinical centers: Besançon: Catherine Chirouze, Elodie Curlier, Cécile Descottes-Genon, Bruno Hoen, Isabelle Patry, Lucie Vettoretti. Dijon: Pascal Chavanet, Jean-Christophe Eicher, Marie-Christine Greusard, Catherine Neuwirth, André Péchinot, Lionel Piroth. Lyon: Marie Célard, Catherine Cornu, François Delahaye, Malika Hadid, Pascale Rausch. Montpellier: Audrey Coma, Florence Galtier, Philippe Géraud, Hélène Jean-Pierre, Vincent Le Moing, Catherine Sportouch, Jacques Reynes. Nancy: Nejla Aissa, Thanh Doco-Lecompte, François Goehringer, Nathalie Keil, Lorraine Letranchant, Hepher Malela, Thierry May, Christine Selton-Suty. Nîmes: Nathalie Bedos, Jean-Philippe Lavigne, Catherine Lechiche, Albert Sotto. Paris: Xavier Duval, Emila Ilic Habensus, Bernard Iung, Catherine Leport, Pascale Longuet, Raymond Ruimy. Rennes: Eric Bellissant, Pierre-Yves Donnio, Fabienne Le Gac, Christian Michelet, Matthieu Revest, Pierre Tattevin, Elise Thebault.

Coordination and statistical analyses: François Alla, Pierre Braquet, Sébastien Dufour, MarieLine Erpelding, Laetitia Minary, Sarah Tubiana.

Centre National de Référence des Staphylocoques: Michèle Bès, Coralie Bouchiat, Jérôme Etienne, Karen Moreau, Anne Tristan, François Vandenesch.

Erasmus University Rotterdam: Alex van Belkum, Willem Van Wamel.

Sponsor CHU de Montpellier: Sandrine Barbas, Christine Delonca, Virginie Sussmuth, Anne Verchère.

### DANSAB Study Group

Herlev: Magnus Arpi, Rigshospitalet: Helle Krogh Johansen, Christian Johann Lerche, Christian Hassager, Henrik Ullum, Erik Sørensen. Aalborg : Eva Korup, Henrik Carl Schoenheyder, Christian Torp-Pedersen. Odense: Flemming S. Rosenvinge, Sabine Gill. Aarhus: Lise Tornvig Erikstrup. Statens Serum Institut: Anders Rhod Larsen, Andreas Petersen.

### COFRASA Study Group

We would like to specially thank Ms Isabelle Codogno for her assistance, the team of the Centre d’Investigation Clinique, Christophe Aucan from the Assistance Publique – Hôpitaux de Paris Département de la Recherche Clinique et du Développement (DRCD) and Estelle Marcault from the Unité de Recherche Clinique Paris Nord for their help and support during all these years.

## Ethics Statement

The VIRSTA study was carried out in accordance with the recommendations of French national ethics committee “Comité de Protection des Personnes SudMéditerrannée IV” with written informed consent from all subjects in accordance with the Declaration of Helsinki. The protocol was approved by the French national ethics committee “Comité de Protection des Personnes SudMéditerrannée IV”. COFRASA participants were enrolled under procedures approved by the “Comité de Protection de Personnes,” Hotel Dieu, Paris, France. The procedure is registered in clinicalTrial.gov number NCT 00338676. DANSAB participants were enrolled under procedures approved by the Danish Regional Ethics Committee (Journal No. H-4-2014-132) and the Danish Data Protection Agency (GEH-2014-053 // I-suite no 03372 and journal no. 2007-58-0015). For eQTL study, written informed consent was obtained from all study participants and the study was approved by the ethics committee of the IUCPQ.

## Author Contributions

VLM, XD, AvB, and FV contributed conception and design of the study. FC performed PCR and sequence analysis. ME extracted and analyzed clinical data of the VIRSTA cohort. AC and YB performed the statistical analysis and wrote sections of the manuscript. PA, NB, RS, CB, KM, and FV conceived the replication study. PA, NB, and RS collected data from the DANSAB cohort and contributed to a fruitful discussion. DM-Z collected data from COFRASA. KM and FV coordinated the project, collected the data, and wrote the first draft of the manuscript. All authors contributed to manuscript revision and read and approved the submitted version.

## Conflict of Interest Statement

AvB is a bioMerieux employee but the company had no influence on the design of the study and the analysis of the data. The other authors declare that the research was conducted in the absence of any commercial or financial relationships that could be construed as a potential conflict of interest. The reviewer NC and handling Editor declared their shared affiliation.

## Footnotes

1 www.glimdna.org

## References

[B1] 1000 Genomes Project Consortium Auton A. Brooks L. D. Durbin R. M. Garrison E. P. Kang H. M. (2015). A global reference for human genetic variation. *Nature* 526 68–74. 10.1038/nature15393 26432245 PMC4750478

[B2] Athanasiadis G. Cheng J. Y. Vilhjálmsson B. J. Jørgensen F. G. Als T. D. Le Hellard S. (2016). Nationwide genomic study in Denmark reveals remarkable population homogeneity. *Genetics* 204 711–722. 10.1534/genetics.116.189241 27535931 PMC5068857

[B3] Bannay A. Hoen B. Duval X. Obadia J.-F. Selton-Suty C. Le Moing V. (2011). The impact of valve surgery on short- and long-term mortality in left-sided infective endocarditis: do differences in methodological approaches explain previous conflicting results? *Eur. Heart J.* 32 2003–2015. 10.1093/eurheartj/ehp008 19208650

[B4] Barrett J. C. Fry B. Maller J. Daly M. J. (2005). Haploview: analysis and visualization of LD and haplotype maps. *Bioinformatics* 21 263–265. 10.1093/bioinformatics/bth457 15297300

[B5] Bouchiat C. Moreau K. Devillard S. Rasigade J.-P. Mosnier A. Geissmann T. (2015). *Staphylococcus aureus* infective endocarditis versus bacteremia strains: subtle genetic differences at stake. *Infect. Genet. Evol.* 36 524–530. 10.1016/j.meegid.2015.08.029 26318542

[B6] Bustamante J. Tamayo E. Flórez S. Telleria J. J. Bustamante E. López J. (2011). Toll-like receptor 2 R753Q polymorphisms are associated with an increased risk of infective endocarditis. *Rev. Esp. Cardiol.* 64 1056–1059. 10.1016/j.recesp.2011.02.024 21783307

[B7] Cabell C. H. Jollis J. G. Peterson G. E. Corey G. R. Anderson D. J. Sexton D. J. (2002). Changing patient characteristics and the effect on mortality in endocarditis. *Arch. Intern. Med.* 162 90–94. 10.1001/archinte.162.1.90 11784225

[B8] Chang F.-Y. MacDonald B. B. Peacock J. E. Musher D. M. Triplett P. Mylotte J. M. (2003). A prospective multicenter study of *Staphylococcus aureus* bacteremia: incidence of endocarditis, risk factors for mortality, and clinical impact of methicillin resistance. *Medicine* 82 322–332. 10.1097/01.md.0000091185.93122.40 14530781

[B9] Cyr D. D. Allen A. S. Du G.-J. Ruffin F. Adams C. Thaden J. T. (2017). Evaluating genetic susceptibility to *Staphylococcus aureus* bacteremia in African Americans using admixture mapping. *Genes Immun.* 18 95–99. 10.1038/gene.2017.6 28332560 PMC5435963

[B10] del Rio A. Cervera C. Moreno A. Moreillon P. Miró J. M. (2009). Patients at risk of complications of *Staphylococcus aureus* bloodstream infection. *Clin. Infect. Dis.* 48(Suppl. 4), S246–S253. 10.1086/598187 19374580

[B11] DeLorenze G. N. Nelson C. L. Scott W. K. Allen A. S. Ray G. T. Tsai A.-L. (2016). Polymorphisms in HLA Class II genes are associated with susceptibility to *Staphylococcus aureus* infection in a white population. *J. Infect. Dis.* 213 816–823. 10.1093/infdis/jiv483 26450422 PMC4747615

[B12] Du P. Kibbe W. A. Lin S. M. (2008). Lumi: a pipeline for processing Illumina microarray. *Bioinformatics* 24 1547–1548. 10.1093/bioinformatics/btn224 18467348

[B13] Duval X. Caplanusi A. Laurichesse H. Deplanque D. Loulergue P. Vaman T. (2012). Flexibility of interval between vaccinations with AS03A-adjuvanted influenza A (H1N1) 2009 vaccine in adults aged 18-60 and > 60 years: a randomized trial. *BMC Infect. Dis.* 12:162. 10.1186/1471-2334-12-162 22824474 PMC3522029

[B14] Embil J. Ramotar K. Romance L. Alfa M. Conly J. Cronk S. (1994). Methicillin resistant *Staphylococcus aureus* in tertiary care institutions on the Canadian prairies 19901992. *Infect. Control Hosp. Epidemiol.* 15 646–651. 10.2307/30145275 7844335

[B15] Emonts M. Uitterlinden A. G. Nouwen J. L. Kardys I. Maat M. P. M. de Melles D. C. (2008). Host polymorphisms in interleukin 4, complement factor H, and C-reactive protein associated with nasal carriage of *Staphylococcus aureus* and occurrence of boils. *J. Infect. Dis.* 197 1244–1253. 10.1086/533501 18422436

[B16] Erichsen P. Gislason G. H. Bruun N. E. (2016). The increasing incidence of infective endocarditis in Denmark, 1994-2011. *Eur. J. Intern. Med.* 35 95–99. 10.1016/j.ejim.2016.05.021 27339641

[B17] Gebo K. A. Burkey M. D. Lucas G. M. Moore R. D. Wilson L. E. (2006). Incidence of, risk factors for, clinical presentation, and 1-year outcomes of infective endocarditis in an urban HIV cohort. *J. Acquir. Immune Defic. Syndr.* 43 426–432. 10.1097/01.qai.0000243120.67529.78 17099314

[B18] Giannitsioti E. Damoraki G. Rokkas C. Tsaganos T. Fragou A. Kannelaki S. (2014). Impact of haplotypes of TNF in the natural course of infective endocarditis. *Clin. Microbiol. Infect.* 20 459–464. 10.1111/1469-0691.12370 24165416

[B19] Golovkin A. S. Ponasenko A. V. Yuzhalin A. E. Salakhov R. R. Khutornaya M. V. Kutikhin A. G. (2015). An association between single nucleotide polymorphisms within TLR and TREM-1 genes and infective endocarditis. *Cytokine* 71 16–21. 10.1016/j.cyto.2014.08.001 25213166

[B20] Guauque-Olarte S. Messika-Zeitoun D. Droit A. Lamontagne M. Tremblay-Marchand J. Lavoie-Charland E. (2015). Calcium signaling pathway genes RUNX2 and CACNA1C are associated with calcific aortic valve disease. *Circ. Cardiovasc. Genet.* 8 812–822. 10.1161/CIRCGENETICS.115.001145 26553695 PMC4934886

[B21] Habib G. Hoen B. Tornos P. Thuny F. Prendergast B. Vilacosta I. (2009). Guidelines on the prevention, diagnosis, and treatment of infective endocarditis (new version 2009): the Task Force on the Prevention, Diagnosis, and Treatment of Infective Endocarditis of the European Society of Cardiology (ESC). *Eur. Heart J.* 30 2369–2413. 10.1093/eurheartj/ehp285 19713420

[B22] Hill P. C. Birch M. Chambers S. Drinkovic D. Ellis-Pegler R. B. Everts R. (2001). Prospective study of 424 cases of *Staphylococcus aureus* bacteraemia: determination of factors affecting incidence and mortality. *Intern. Med. J.* 31 97–103. 10.1111/j.1444-0903.2001.00029.x 11480485

[B23] Hindorff L. A. Sethupathy P. Junkins H. A. Ramos E. M. Mehta J. P. Collins F. S. (2009). Potential etiologic and functional implications of genome-wide association loci for human diseases and traits. *Proc. Natl. Acad. Sci. U.S.A.* 106 9362–9367. 10.1073/pnas.0903103106 19474294 PMC2687147

[B24] Hoen B. Duval X. (2012). Epidemiology of infective endocarditis. *Rev. Prat.* 62 511–514. 22641893

[B25] Jin Z.-B. Huang X.-F. Lv J.-N. Xiang L. Li D.-Q. Chen J. (2014). SLC7A14 linked to autosomal recessive retinitis pigmentosa. *Nat. Commun.* 5:3517. 10.1038/ncomms4517 24670872 PMC3974215

[B26] Kallen A. J. Reed C. Patton M. Arnold K. E. Finelli L. Hageman J. (2010). *Staphylococcus aureus* community-onset pneumonia in patients admitted to children’s hospitals during autumn and winter of 2006-2007. *Epidemiol. Infect.* 138 666–672. 10.1017/S095026880999135X 19961644

[B27] Klevens R. M. Morrison M. A. Nadle J. Petit S. Gershman K. Ray S. (2007). Invasive methicillin-resistant *Staphylococcus aureus* infections in the United States. *JAMA* 298 1763–1771. 10.1001/jama.298.15.1763 17940231

[B28] Laupland K. B. (2013). Incidence of bloodstream infection: a review of population-based studies. *Clin. Microbiol. Infect.* 19 492–500. 10.1111/1469-0691.12144 23398633

[B29] Le Moing V. Alla F. Doco-Lecompte T. Delahaye F. Piroth L. Chirouze C. (2015). *Staphylococcus aureus* bloodstream infection and endocarditis–A prospective cohort study. *PLoS One* 10:e0127385. 10.1371/journal.pone.0127385 26020939 PMC4447452

[B30] Lek M. Karczewski K. J. Minikel E. V. Samocha K. E. Banks E. Fennell T. (2016). Analysis of protein-coding genetic variation in 60,706 humans. *Nature* 536 285–291. 10.1038/nature19057 27535533 PMC5018207

[B31] Li J. S. Sexton D. J. Mick N. Nettles R. Fowler V. G. Ryan T. (2000). Proposed modifications to the Duke criteria for the diagnosis of infective endocarditis. *Clin. Infect. Dis.* 30 633–638. 10.1086/313753 10770721

[B32] Li W. Somerville J. (1998). Infective endocarditis in the grown-up congenital heart (GUCH) population. *Eur. Heart J.* 19 166–173. 10.1053/euhj.1997.0821 9503191

[B33] Maguire G. P. Arthur A. D. Boustead P. J. Dwyer B. Currie B. J. (1998). Clinical experience and outcomes of community-acquired and nosocomial methicillin-resistant *Staphylococcus aureus* in a northern Australian hospital. *J. Hosp. Infect.* 38 273–281. 10.1016/S0195-6701(98)90076-7 9602976

[B34] Medvedev A. E. Vogel S. N. (2003). Overexpression of CD14, TLR4, and MD-2 in HEK 293T cells does not prevent induction of in vitro endotoxin tolerance. *J. Endotoxin Res.* 9 60–64. 10.1179/096805103125001360 12691621

[B35] Miro J. M. Anguera I. Cabell C. H. Chen A. Y. Stafford J. A. Corey G. R. (2005). *Staphylococcus aureus* native valve infective endocarditis: report of 566 episodes from the International Collaboration on Endocarditis Merged Database. *Clin. Infect. Dis.* 41 507–514. 10.1086/431979 16028160

[B36] Moreillon P. Que Y.-A. (2004). Infective endocarditis. *Lancet* 363 139–149. 10.1016/S0140-6736(03)15266-X 14726169

[B37] Murdoch D. R. Corey G. R. Hoen B. Miró J. M. Fowler V. G. Bayer A. S. (2009). Clinical presentation, etiology, and outcome of infective endocarditis in the 21st century: the International Collaboration on Endocarditis-Prospective Cohort Study. *Arch. Intern. Med.* 169 463–473. 10.1001/archinternmed.2008.603 19273776 PMC3625651

[B38] Nelson C. L. Pelak K. Podgoreanu M. V. Ahn S. H. Scott W. K. Allen A. S. (2014). A genome-wide association study of variants associated with acquisition of *Staphylococcus aureus* bacteremia in a healthcare setting. *BMC Infect. Dis.* 14:83. 10.1186/1471-2334-14-83 24524581 PMC3928605

[B39] Nguyen V. Cimadevilla C. Estellat C. Codogno I. Huart V. Benessiano J. (2015). Haemodynamic and anatomic progression of aortic stenosis. *Heart* 101 943–947. 10.1136/heartjnl-2014-307154 25655063

[B40] Oestergaard L. B. Christiansen M. N. Schmiegelow M. D. Skov R. L. Andersen P. S. Petersen A. (2016). Familial clustering of *Staphylococcus aureus* Bacteremia in first-degree relatives: a Danish nationwide cohort study. *Ann. Intern. Med.* 165 390–398. 10.7326/M15-2762 27379577

[B41] Pe’er I. Yelensky R. Altshuler D. Daly M. J. (2008). Estimation of the multiple testing burden for genomewide association studies of nearly all common variants. *Genet. Epidemiol.* 32 381–385. 10.1002/gepi.20303 18348202

[B42] Picard C. Puel A. Bonnet M. Ku C.-L. Bustamante J. Yang K. (2003). Pyogenic bacterial infections in humans with IRAK-4 deficiency. *Science* 299 2076–2079. 10.1126/science.1081902 12637671

[B43] Ponasenko A. V. Kutikhin A. G. Khutornaya M. V. Rutkovskaya N. V. Kondyukova N. V. Odarenko Y. N. (2017). Inherited variation in cytokine, acute phase response, and calcium metabolism genes affects susceptibility to infective endocarditis. *Mediators Inflamm.* 2017 1–21. 10.1155/2017/7962546 28659664 PMC5474236

[B44] Price A. L. Patterson N. J. Plenge R. M. Weinblatt M. E. Shadick N. A. Reich D. (2006). Principal components analysis corrects for stratification in genome-wide association studies. *Nat. Genet.* 38 904–909. 10.1038/ng1847 16862161

[B45] Purcell S. Neale B. Todd-Brown K. Thomas L. Ferreira M. A. R. Bender D. (2007). PLINK: a tool set for whole-genome association and population-based linkage analyses. *Am. J. Hum. Genet.* 81 559–575. 10.1086/519795 17701901 PMC1950838

[B46] Rasmussen R. V. Høst U. Arpi M. Hassager C. Johansen H. K. Korup E. (2011). Prevalence of infective endocarditis in patients with *Staphylococcus aureus* bacteraemia: the value of screening with echocardiography. *Eur. J. Echocardiogr.* 12 414–420. 10.1093/ejechocard/jer023 21685200 PMC3117467

[B47] Ruimy R. Angebault C. Djossou F. Dupont C. Epelboin L. Jarraud S. (2010). Are host genetics the predominant determinant of persistent nasal *Staphylococcus aureus* carriage in humans? *J. Infect. Dis.* 202 924–934. 10.1086/655901 20677941

[B48] Selton-Suty C. Célard M. Le Moing V. Doco-Lecompte T. Chirouze C. Iung B. (2012). Preeminence of *Staphylococcus aureus* in infective endocarditis: a 1-year population-based survey. *Clin. Infect. Dis.* 54 1230–1239. 10.1093/cid/cis199 22492317

[B49] Takeda S. Nakanishi T. Nakazawa M. (2005). A 28-year trend of infective endocarditis associated with congenital heart diseases: a single institute experience. *Pediatr. Int.* 47 392–396. 10.1111/j.1442-200x.2005.02076.x 16091075

[B50] Telenti A. Pierce L. C. T. Biggs W. H. di Iulio J. Wong E. H. M. Fabani M. M. (2016). Deep sequencing of 10,000 human genomes. *Proc. Natl. Acad. Sci. U.S.A.* 113 11901–11906. 10.1073/pnas.1613365113 27702888 PMC5081584

[B51] Tubiana S. Duval X. Alla F. Selton-Suty C. Tattevin P. Delahaye F. (2016). The VIRSTA score, a prediction score to estimate risk of infective endocarditis and determine priority for echocardiography in patients with *Staphylococcus aureus* bacteremia. *J. Infect.* 72 544–553. 10.1016/j.jinf.2016.02.003 26916042

[B52] Warren B. A. Yong J. L. (1997). Calcification of the aortic valve: its progression and grading. *Pathology* 29 360–368. 10.1080/00313029700169315 9423215

[B53] Weinstock M. Grimm I. Dreier J. Knabbe C. Vollmer T. (2014). Genetic variants in genes of the inflammatory response in association with infective endocarditis. *PLoS One* 9:e110151. 10.1371/journal.pone.0110151 25299518 PMC4192365

[B54] Ye Z. Vasco D. A. Carter T. C. Brilliant M. H. Schrodi S. J. Shukla S. K. (2014). Genome wide association study of SNP-, gene-, and pathway-based approaches to identify genes influencing susceptibility to Staphylococcus aureus infections. *Front. Genet.* 5:125. 10.3389/fgene.2014.00125 24847357 PMC4023021

