# Supplementary material for: Human Genetic Susceptibility to Native Valve Staphylococcus aureus Endocarditis in Patients With S. aureus Bacteremia: Genome-Wide Association Study
Source: Front Microbiol. 2018 Apr 4;9:640. doi: 10.3389/fmicb.2018.00640 (PMC5893849; doi:10.3389/fmicb.2018.00640)
Supplement: Supplementary file 1 [file Image_1.pdf]

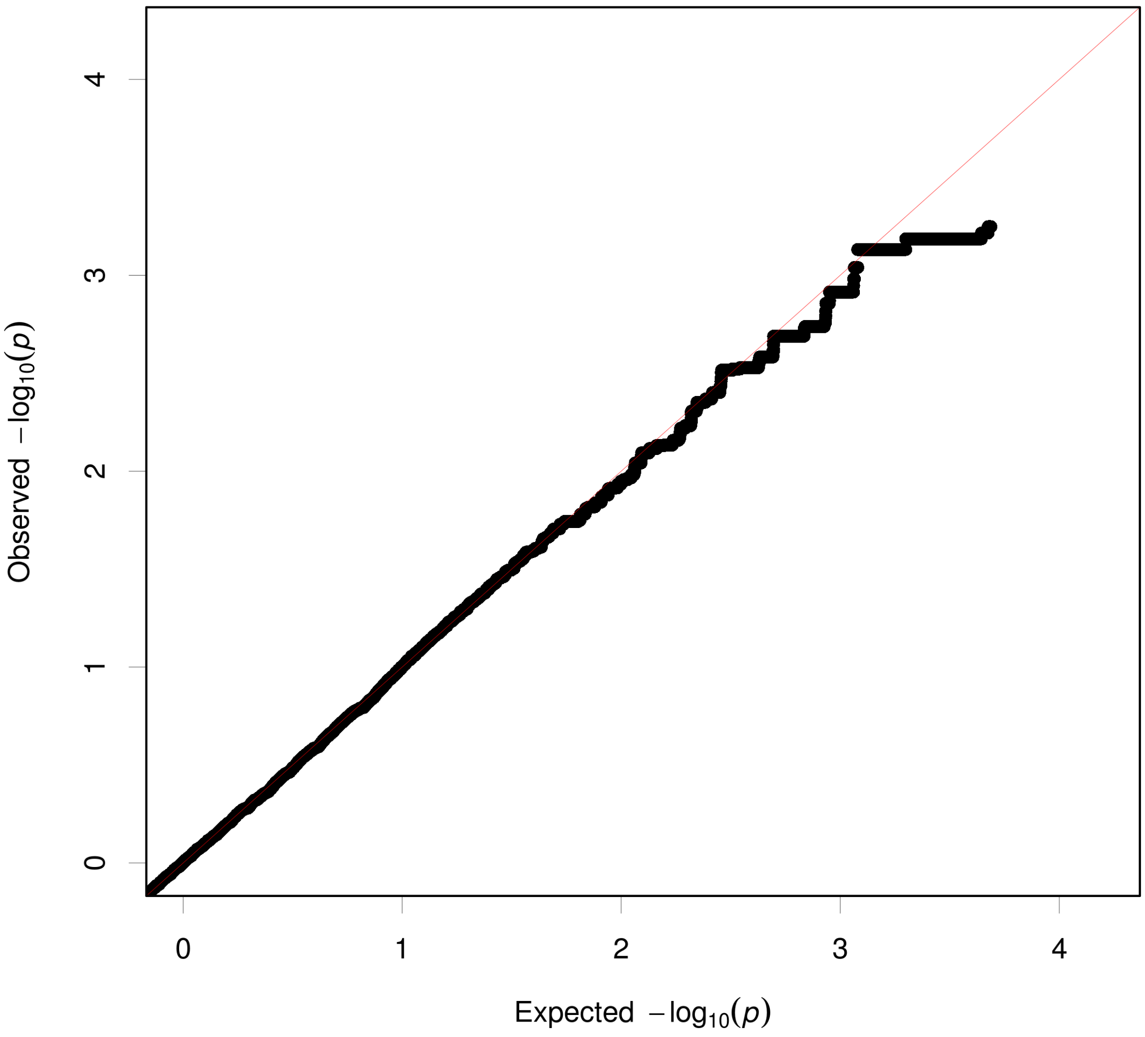

Supplementary Fig 1. Quantile-quantile plot of test statistics generated by the GWAS in 67 cases and 72 controls including 631,710 SNPs.
